# Literary Notes

**Published:** 1903-12

**Authors:** 


### LITERARY NOTES.

THE *Daily Medical Journal* announces that, owing to delay in securing European correspondents, its publication is postponed to January 1, 1904.

---

THE *Journal of Tuberculosis*, heretofore a quarterly, will be published as a monthly, beginning January 15, 1904. Some improvements in the magazine will be made, but the subscription price will remain the same—$3.00.

The Enno Sander Prize.—The essayist securing first place will receive a gold medal of the value of $100.00. The essayist securing second place will receive a life membership in the Association of Military Surgeons of the United States of the value of $50.00. Subject of the competition for 1904 is, The relation of the medical department to the health of armies.

Among the conditions of the competition are that it is open to all persons eligible to active or associate membership in the Association of Military Surgeons of the United States; that essays will consist of not less than ten thousand, nor more than twelve thousand words, exclusive of tables; that each competitor will send three typewritten copies of his essay in a sealed envelope to the secretary of the association, so as to reach that officer *at least one month before the next ensuing annual meeting,* in the present case on or before September 10, 1904.

The board of award is composed of Lieutenant Colonel John Shaw Billings, U. S. Army; Brevet Brigadier General George Ryerson Fowler, New York and Surgeon Henry Gustav Beyer, U. S. Navy.

---

E. B. Treat & Co., announce the following books in press: The Blues. (splanchnic neurasthenia), causes and cure. By Albert Abrams, M. D., F. R. M. S. Octavo, 230 pages, illustrated, $1.25. Diseases of Metabolism and Nutrition. Part IV. Autointoxication. By Prof. Dr. Carl von Noorden and Dr. Mohr. Authorised American edition. Edited by Boardman Reed, M. D. Small 8vo; 80 pages, 50 cents.

